# Adipogenic progenitors keep muscle stem cells young

**DOI:** 10.18632/aging.102304

**Published:** 2019-09-19

**Authors:** Sara Ancel, Omid Mashinchian, Jerome N. Feige

**Affiliations:** 1 Nestlé Research, EPFL Innovation Park, Lausanne, Switzerland; 2 School of Life Sciences, Ecole Polytechnique Fédérale de Lausanne (EPFL), Switzerland

**Keywords:** muscle stem cell, regeneration, aging, fibro/adipogenic progenitor, niche

Declining stem cell function during aging leads to impaired tissue function and contributes to delayed tissue repair following damage. In adult skeletal muscle, loss of myofiber integrity caused by mechanical injuries or diseases are repaired by resident muscle stem cells (MuSCs), called satellite cells, which promptly exit from quiescence after disruption of muscle architecture to expand, differentiate and drive tissue regeneration. The fate of MuSCs fundamentally depends on the “niche”, their local environment, which is orchestrated by diverse cellular and acellular elements. Aging causes cell-extrinsic changes to the MuSC niche and dysregulates signaling pathways, which collectively alter the regenerative function of MuSCs [1]. The low regenerative capacity of aged muscle also contributes to the development of fibrosis in response to aberrant systemic cues.

**Figure 1:**
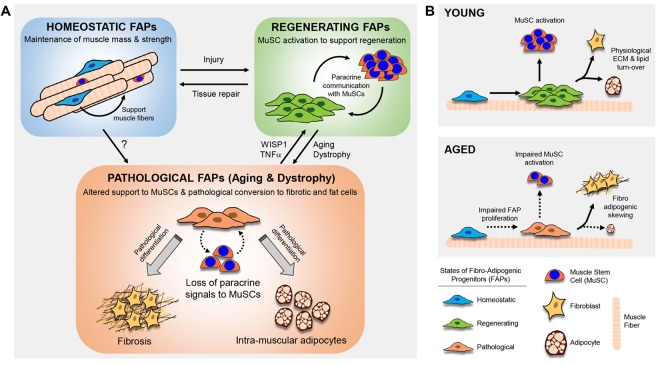
**Fibro-adipogenic progenitors (FAPs) dynamically cross-talk with the muscle stem cell (MuSC) niche to regulate regeneration and ECM/lipid turnover in different patho-physiologcal cues.** (**A**) FAPs have distinct cellular fates and functions in different patho-physiological conditions; support to muscle fibers in homeostatic conditions [5]; support to muscle stem cells during regeneration [2]; pathological differentiation to fat and fibrosis [4,6]. (**B**) Aging alters the support of FAPs to MuSCs and regeneration and promotes their pathological skewing to fibrosis over adipogenesis [6].

Fibro/adipogenic progenitors (FAPs) constitute a population of interstitial mesenchymal cells in skeletal muscle which are devoid of myogenic potential, but support muscle stem cell commitment and can differentiate to the adipogenic or fibrotic lineages (Figure 1A) [2]. A “regenerative” population of FAPs expands in response to injury to supply transient support-signals and regulate the commitment of MuSCs during muscle regeneration [3,4]. Genetic muscle diseases such as Duchenne muscular dystrophy generate “pathological” FAPs, and cause dysbiosis between FAPs and MuSCs, impaired regenerative capacity and muscle infiltration of adipose tissue and fibrosis [4]. However, much less is known on the chronic contribution of FAPs to muscle homeostasis and on the influence of aging on the cross-talk between FAPs and MuSCs.

A recent study from the Rando laboratory demonstrated an important function of FAPs in maintaining long-term homeostasis of skeletal muscle [5]. Long term *in-vivo* depletion of “homeostatic” FAPs using PDGFRα-Cre mediated ablation decreased the number of MuSCs and reduced muscle mass and strength, suggesting a critical role of FAPs in maintaining the stem cell pool and sustaining myofiber growth and turnover. In a more acute setting, the absence of regenerative FAP amplification during regeneration following muscle injury also blocked MuSC expansion and delayed regeneration of damaged myofibers. Interestingly, FAP depletion also impaired expansion of CD45+ hematopoietic cells at the site of injury [5]. Thus, FAPs are active regulators of cellular communication in skeletal muscle niche where they directly control tissue homeostasis and regeneration by supporting MuSCs and myofibers.

The decline of MuSC function and muscle regenerative capacity during aging is under the control of a wide range of signals, out of which many arise from extrinsic cues coming from the local or systemic environment [1]. In a recent study, our lab investigated how aging influences the fate of FAPs and their cross-talk with MuSCs to regulate the balance between myogenesis, adipogenesis and fibrosis in skeletal muscle ([6]; Figure 1B). Aging causes a clonal selection of FAPs, which favors their fibrogenic over adipogenic conversion. Interestingly, aged FAPs fail to efficiently amplify following muscle injury and aging alters the capacity of FAPs to support MuSC amplification and commitment. Both *in-vitro* co-culture and *in-vivo* transplantation of young FAPs rejuvenate aged MuSC function, but aged FAPs lose the ability to efficiently support MuSCs. The fact that the support of FAPs to MuSCs is communicable via conditioned medium suggested that soluble factors regulate this paracrine cross-talk. Using transcriptomic profiling followed by *in-vitro* and *in-vivo* validation, we could identify that altered secretion of the FAP-secreted matricellular protein WISP-1 (WNT1 Inducible Signaling Pathway Protein 1, also called CCN-4) drives the perturbed support of aged FAPs to MuSCs. Genetic invalidation of WISP1 disrupts the communication between FAPs and MuSCs and leads to abnormal regeneration of skeletal muscle. Mechanistically, WISP-1 controls MuSC asymmetric expansion by activating Akt signaling. Importantly, this new paracrine mechanism can be targeted therapeutically by administering recombinant WISP-1 systemically. Altogether, our work reveals that loss of WISP1 from FAPs directly contributes to MuSC dysfunction in aged muscle and demonstrates that adipogenic progenitors are not just quiescent precursors waiting for pathological cues to elicit pathological conversions, but actually constitute bona fide elements of the stem cell niche, which regulate an active cross-talk with stem cells via paracrine signaling [6].

Along similar lines, a subpopulation of stromal progenitors in adipose tissue has recently been identified using single-cell RNA sequencing as a negative regulator of adipogenesis which represses the differentiation of adipocytes and keeps fat expansion in check [7]. FAPs are also likely a heterogeneous population and the clonal selection of different fates of FAPs during aging suggests a differential effect of age on distinct subpopulations. A recent study by Malecova et al. [8] reports a dynamic specialization of sub-FAPs in physiological and diseased conditions. While Tie2-expressing FAPs predominantly reside within neonatal and adult homeostatic muscles, another injury-activated subpopulation of FAPs characterized by Vcam1 expression is associated with regeneration of injured myofibers [8]. Future research will be necessary to further dissect FAP function during homeostasis and tissue repair and unravel how the heterogeneity of this population is orchestrated in health and disease. In particular, the signals that mediate FAP dysfunction and the spatio-temporal control of their fate and interactions with MuSCs will be key to understand how aging of different compartments of the stem cell niche contribute to global regenerative capacity.
